# In Vivo Irritation and Sensitization Assessment of Cypermethrin, Pymetrozine, and Indoxacarb–Emamectin Benzoate Combination in Test Animals

**DOI:** 10.1155/jt/1282928

**Published:** 2025-08-25

**Authors:** Wahyu Daradjat Natawigena, Kirana Fayruz Swarga, Gloria Mahayarni Lastiar Sitinjak, Muhammad Ilfadry Rifasta, Agus Susanto, Gofarana Wilar, Cecep Suhandi

**Affiliations:** ^1^Department of Plant Pests and Diseases, Faculty of Agriculture, Padjadjaran University, Sumedang 45363, Indonesia; ^2^Pharmacy Undergraduate Study Program, Faculty of Pharmacy, Padjadjaran University, Sumedang 45363, Indonesia; ^3^Department of Pharmacology and Clinical Pharmacy, Faculty of Pharmacy, Padjadjaran University, Sumedang 45363, Indonesia

**Keywords:** cypermethrin, eye irritation, indoxacarb–emamectin benzoate, pymetrozine, skin irritation, skin sensitization

## Abstract

Insecticides are widely used to protect agricultural and plantation products from pests and plant-disrupting organisms. Prior to market distribution, pesticide-containing products must undergo safety evaluations, including assessments of potential skin and eye irritation and allergic responses. In this study, we conducted one of the first comparative in vivo evaluations of commercial insecticidal formulations containing cypermethrin (Gusano 300 EC), pymetrozine (Vorum 50 WG), and indoxacarb–emamectin benzoate (Endorse Plus 160/20 OD) using internationally recognized protocols based on OECD 404 (skin irritation), OECD 405 (eye irritation), and OECD 406 (skin sensitization). The results showed that the cypermethrin formulation caused minimal skin irritation, mild eye irritation, and skin sensitization. The pymetrozine formulation did not cause skin irritation or sensitization but induced mild eye irritation. The indoxacarb–emamectin Benzoate formulation caused mild eye irritation and skin sensitization, with minimal skin irritation. These findings contribute novel data to the toxicological profile of these pesticide formulations and support future safety evaluations and regulatory assessments.

## 1. Introduction

Pesticides are distinctive environmental contaminants aimed at preventing, eliminating, repelling, or mitigating pests, disease vectors, or unwanted vegetation that can disrupt the quality of agricultural and plantation products [1, 2]. These substances include insecticides, fungicides, herbicides, rodenticides, molluscicides, and nematicides [3, 4]. Insecticides, in particular, are further divided into synthetic and natural types, each with its advantages and limitations [4]. Synthetic insecticides are chemically categorized into organophosphates, carbamates, organochlorines, and pyrethroids [5, 6].

Pyrethroids are artificial insecticides divided into two types: Type I (without a cyano moiety) and Type II (with a cyano moiety). Type II pyrethroids, such as cypermethrin, fenvalerate, and deltamethrin, are known for their improved efficacy and environmental persistence [7]. Cypermethrin is a potent, broad-spectrum insecticide widely used in agriculture and domestic applications [8]. It works by prolonging the opening of voltage-gated sodium channels in nerve cells, leading to repetitive nerve firing, paralysis, and death in insects. However, in mammals, topical exposure to cypermethrin has been reported to cause skin and eye irritation, likely due to localized inflammatory responses, disruption of cell membranes, and oxidative stress in epithelial tissues [9].

Pymetrozine is a pyridine azomethine derivative that selectively targets the feeding behavior of sap-sucking insects such as aphids and whiteflies [10]. It acts systemically by interfering with chordotonal organ function, disrupting neural signal transduction related to feeding [11]. While generally considered to have low acute toxicity in mammals, its formulation or residues may still cause eye or skin irritation, especially when applied in high concentrations or over prolonged periods. Mechanistically, its surfactants or coformulants may compromise skin barrier integrity or induce subclinical inflammation [12].

Indoxacarb, a pyrazoline-type insecticide, blocks sodium channels in insects, leading to paralysis and death. It is commonly formulated with emamectin benzoate for synergistic insecticidal activity. In mammals, although its systemic toxicity is relatively low, chronic exposure to indoxacarb has been associated with oxidative tissue damage in aquatic models [13]. Moreover, its emulsifiable concentrates may act as mild irritants due to solvent components or excipients. Emamectin benzoate, derived from avermectin, can affect gamma-aminobutyric acid (GABA) receptors and may cause transient eye or skin irritation on direct contact [14].

The overuse or improper handling of insecticides can lead to significant public health concerns, including dermal reactions and mucosal irritation, particularly among agricultural workers [15]. The global burden of pesticide poisoning remains high, with an estimated 385 million cases of acute pesticide poisoning annually, and a large proportion of these involve dermal or ocular routes of exposure [16, 17]. Despite the widespread use of commercial insecticide formulations, limited data exist regarding the local irritant and sensitization effects of specific active ingredients such as cypermethrin (Gusano 300 EC), pymetrozine (Vorum 50 WG), and combined indoxacarb–emamectin benzoate (Endorse Plus 160/20 OD). Most previous studies have focused on systemic toxicity rather than localized effects on skin or eyes.

Therefore, the present study aims to evaluate the potential of these insecticide formulations to cause skin irritation, eye irritation, and skin sensitization using in vivo animal models. Skin and eye irritation tests will be conducted using New Zealand white rabbits, while skin sensitization will be assessed in Dunkin–Hartley guinea pigs. The results are expected to provide valuable toxicological data for safety evaluations of these products, particularly for occupational and environmental risk assessments.

## 2. Materials and Methods

### 2.1. Materials

In this study, male New Zealand rabbits weighing between 1.2 and 2.5 kg at 17 weeks of age were procured from the Faculty of Animal Husbandry Universitas Padjadjaran, while male Dunkin–Hartley guinea pigs weighing between 250 and 750 g at 4–6 weeks of age were procured from PT Bio Farma (Persero). Insecticides containing cypermethrin at a concentration of 300 g/L, a combination of indoxacarb at a concentration of 160 g/L and emamectin benzoate at a concentration of 20 g/L, and pymetrozine at a concentration of 50% were procured from the market.

### 2.2. Skin Irritation Test

**Figure 1:**
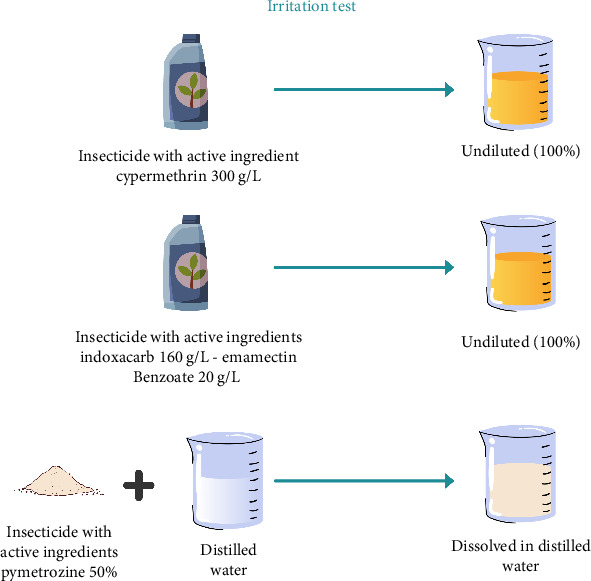
Preparation of test solution, irritation test.

**Figure 2:**
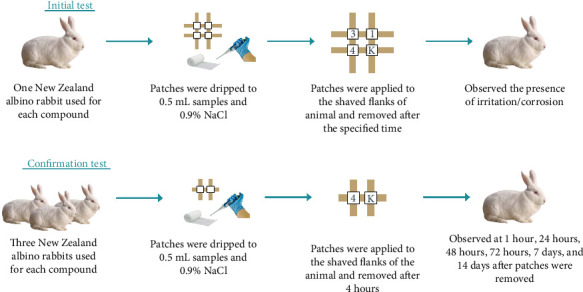
Procedure for skin irritation test.

Insecticides with active ingredients cypermethrin and a combination of indoxacarb–emamectin benzoate are undiluted, and pymetrozine is diluted with the smallest volume of water (Figure 1). In the initial test, one eligible New Zealand albino rabbit is selected. Three patches were dripped with 0.5 mL of the sample, while another gauze patch was dripped with 0.9% NaCl as the control. Subsequently, the gauze patches are applied to the shaved flanks of the animals. After 3 minutes, the first patch is taken out. If no significant cutaneous reaction is observed, a second patch is applied to a different area and removed 1 hour and 4 hours later. If an irritant effect is observed in the initial test, the confirmatory test can be run sequentially or simultaneously by exposing three additional animals. Each animal is treated with a single patch that had been dripped with the test chemical and 1 patch that had been dripped with NaCl for 4 h; the patch is removed, and the responses are observed at 1 h, 1 day, 2 days, and 3 days after patch removal (Figure 2) [18].

All physical responses are assessed based on a comprehensive report utilizing the Draize Skin Irritation Test Rating Scale on the skin in Table 1 [18].

Furthermore, from the obtained erythema and edema scores, the primary irritation index (PII) was calculated for each insecticide formulation. The PII was determined based on the OECD 404 guideline by averaging the cumulative irritation scores (sum of erythema and edema scores) observed at 24, 48, and 72 h after application, divided by the number of observation time points (3) and the number of animals tested (3 rabbits per sample). The formula used is as follows [19]:

(1) $$\begin{array}{c} \text{PII}=\frac{\Sigma A+\Sigma B}{\;6\times C}, \end{array}$$

where *A* is the total score of erythema during observation, *B* is the total score of edema during observation, and *C* is the number of animals treated.

### 2.3. Eye Irritation Test

**Figure 3:**
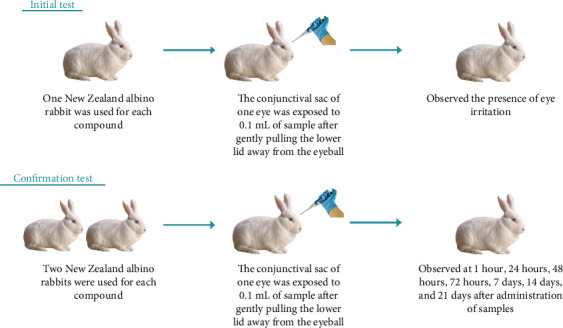
Procedure for eye irritation test.

Insecticides with active ingredients cypermethrin and a combination of indoxacarb–emamectin benzoate are undiluted, and pymetrozine is diluted with the smallest volume of water (Figure 1). In the initial test, one eligible New Zealand albino rabbit was selected and 0.1 mL sample was placed into the conjunctival sac of one eye after gently pulling the lower lid away from the eyeball. If there is no severe eye irritation, we proceed with a confirmation test with 2 additional rabbits. To determine the status and reversibility or irreversibility of lesions, observations should be conducted and recorded at 1 h, 1 day, 2 days, 3 days, 7 days, 14 days, and 21 days (Figure 3) [20].

All physical responses are assessed based on a comprehensive report utilizing the Draize Skin Irritation Test Rating Scale on the skin in Table 2 [20].

### 2.4. Sensitization Test

**Figure 4:**
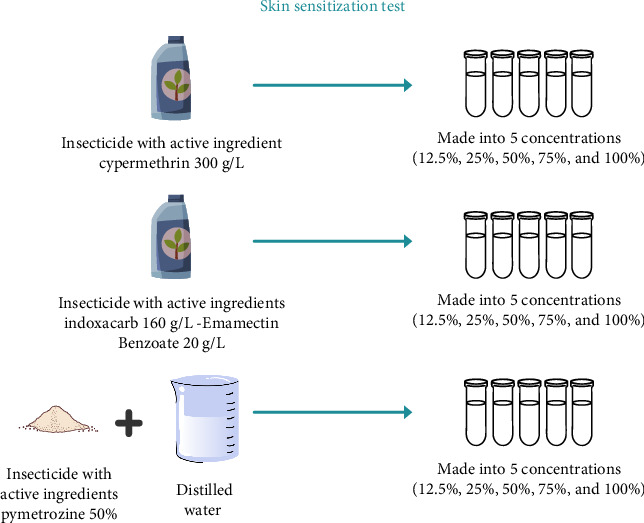
Preparation of test solution, skin sensitization test.

**Figure 5:**
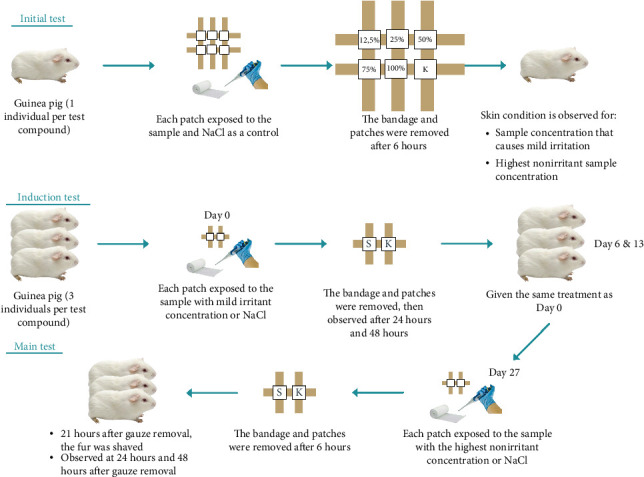
Procedure for skin sensitization test.

Skin sensitization testing refers to OECD 406 using the Buehler test. Initial tests were carried out using one Dunkin–Hartley guinea pig, which was clinically examined with a concentration of 12.5%, 25%, 50%, 70%, and 100%, and NaCl as a control, which was dripped into different patches (Figure 4). The patches were applied to a cleaned test area for 6 hours using a bandage. The main test was then carried out on 3 guinea pigs using the highest concentration that did not cause erythema. Similar treatment was carried out on Days 0, 6, and 13. Then, we continued with the challenge exposure on Day 27; the concentration should be the highest nonirritating dose (Figure 5). Observational data are recorded based on Magnusson and Kligman's rating scale according to Table 3 [21].

## 3. Results and Discussion

### 3.1. Skin Irritation Test

The primary way pesticides enter the body is through the skin, which is the most common route of exposure. As long as pesticides remain in contact with the skin, absorption will continue [22]. Pesticides can cause both systemic and localized reactions on the skin, leading to various clinical effects. When a chemical lingers at the contact site, it can have a residual impact and harm the basal layer of the skin responsible for producing pigment. This can subsequently lead to alterations in the production of epidermal melanin, causing either an increase or decrease in its production [23].

#### 3.1.1. Initial Test

The initial skin irritation test was carried out using 1 rabbit for each sample and observed at Day 0, Day 2, Day 3, Day 7, and Day 14 after treatment. Based on the results of the initial eye irritation test in Table 4., very slight erythema occurred after exposure to cypermethrin on Day 1, Day 2, Day 3, and Day 7 and healed on Day 14. Very slight edema also occurred after exposure to indoxacarb–emamectin benzoate on Day 1, Day 2, Day 3, Day 7, and Day 14. No erythema occurred after exposure to pymetrozine.

Based on the initial test irritation score in Table 4., samples of cypermethrin, pymetrozine, and indoxacarb–emamectin benzoate did not cause an edema formation. To confirm the results of the initial test, the test is continued with a confirmation test.

#### 3.1.2. Confirmation Test

According to the findings presented in Table 5., the erythema that occurred after exposure to the three insecticide samples completely recovered within 14 days, and no significant skin reactions such as skin corrosion were observed. These results align with previous research suggesting that cypermethrin can cause skin irritation in New Zealand albino rabbits [24]. Additionally, pymetrozine exhibited low acute toxicity and was categorized as nonirritating (Toxicity category IV) in a skin irritation test conducted by the United States Environmental Protection Agency [25]. Furthermore, the effects observed with indoxacarb and emamectin benzoate, which are more toxic when ingested orally compared to topically applied, do not qualify as serious skin reactions [26]. It can be observed that after exposure to the three insecticides used in this study, no edema was formed on the rabbit's skin.

Furthermore, from the obtained erythema and edema scores, the PII can be calculated.

**Figure 6:**
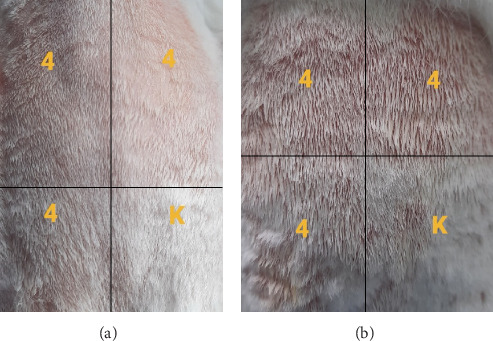
(a) Irritation testing results of an insecticide formula containing the active ingredient cypermethrin on rabbit skin (erythema). (b) Irritation testing results of a compound insecticide formula containing the active ingredients indoxacarb–emamectin benzoate on rabbit skin (erythema).

The results of the PII calculation for each insecticide formula in Table 6 shows that cypermethrin (Gusano 300 EC) at a dose of 0.5 mL applied topically falls into the category of almost nonirritating with an average value of the PII of 0.83 (Figure 6(a)). Pymetrozine (Vorum 50 WG) at a dose of 0.5 mL applied topically falls into the category of nonirritating with an average value of the PII of 0. Furthermore, for the insecticide formula with the combined active ingredients indoxacarb–emamectin benzoate (Endorse Plus 160/20 OD) at a dose of 0.5 mL applied topically, it falls into the category of almost nonirritating with an average value of PII of 0.915 (Figure 6(b)).

### 3.2. Eye Irritation Test

The toxic effects of pesticides can be observed by individuals who are exposed through a variety of entry routes, including the eyes [27]. Eyes are particularly susceptible to absorption; therefore, any contact with pesticides poses an immediate risk of injury, blindness, and occasionally mortality [15]. Eye exposure to pesticides causes damage to the cornea, conjunctiva, lens, retina, and optic nerve, as well as abnormal ocular movement and vision impairment, no reactivity to light/light sensitivity, and excessive tear production [28, 29].

#### 3.2.1. Initial Test

In the initial eye irritation test of cypermethrin, pymetrozine, and indoxacarb–emamectin benzoate samples, one rabbit was used to determine its reversibility. Observations were made at 0 days, 1 day, 2 days, 3 days, 7 days, 14 days, and 21 days after treatment. Based on the results in Table 7, cypermethrin samples experienced a decreased opacity and showed healed on Day 21, swelling of the iris but still reacted to light from Day 0 until Day 3 and healed on Day 7, with no conjunctival reaction. An obvious above-normal condition with lids about half closed occurred on Day 0, swelling with lids about half closed to completely shown on Day 3 and Day 4, and healed on Day 3.

Irritation reaction in the form of swelling on the iris and a slightly above-normal chemosis were shown after exposure to indoxacarb–emamectin benzoate. Exposure to pymetrozine did not cause any irritation reaction. To confirm the results of the initial test, the test is continued with a confirmation test.

#### 3.2.2. Confirmation Test

The confirmation test aims to confirm the irritation reaction of cypermethrin, pymetrozine, and indoxacarb–emamectin benzoate samples that were not observed in the initial test. The test was continued with a confirmation test using 2 rabbits, observed at 0 days, 1 day, 2 days, 3 days, 7 days, 14 days, and 21 days to analyze the irritation index and reversibility.

**Figure 7:**
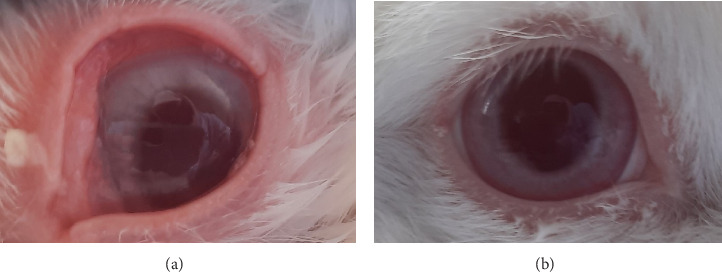
Eye irritation confirmation test in cornea result. (a) Scattered cloudy area on the cornea with clear visibility of iris details. (b) Normal eye.

**Figure 8:**
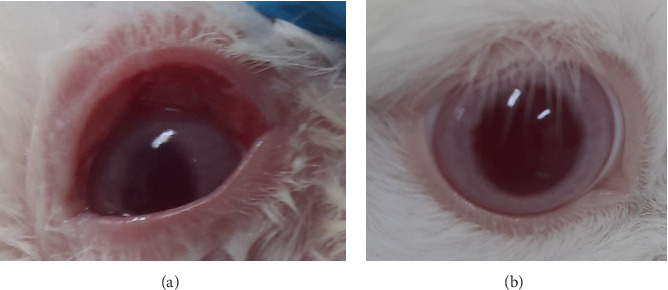
Eye irritation confirmation test in iris result. (a) Swelling of the iris and reactive to light. (b) Normal eye.

**Figure 9:**
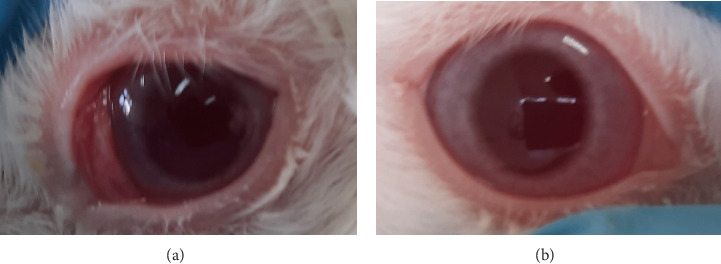
Eye irritation confirmation test in conjunctivae result. (a) Slightly redder than the control eye (hyperemia). (b) Normal eye.

**Figure 10:**
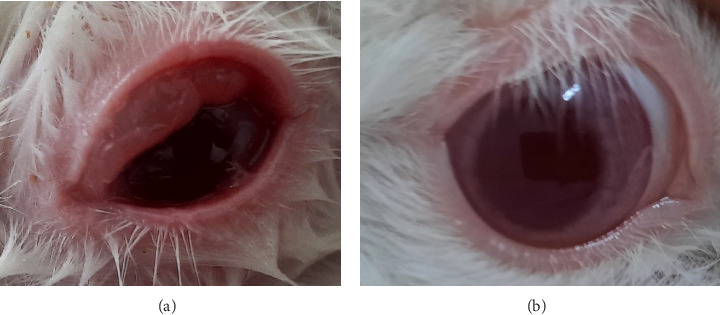
Eye irritation confirmation test in chemosis result. (a) Clear swelling, partial eyelid inversion (chemosis). (b) Normal eye.

Based on the test results presented in Tables 8 and 9 and Figures 7, 8, 9, and 10, the insecticide formula with the single active ingredient cypermethrin (Gusano 300 EC) at a dose of 0.1 mL applied topically to the eyes caused mild irritation in the rabbit's eyes compared to the normal eyes (control), as indicated by corneal opacity (average score = 1.0476), iris swelling (average score = 0.8095), conjunctival redness (average score = 0.0476), and chemosis (average score = 1.1905). However, the eye irritation fully recovered within the observation period of the irritation test, which was 21 days. These test results are supported by the theory that cypermethrin can moderately irritate the eyes of New Zealand albino rabbits [24].

The insecticide formula with the single active ingredient pymetrozine (Vorum 50 WG) at a dose of 0.1 mL which was applied topically to the eyes caused mild irritation to rabbit eyes compared to normal eyes (control), which was characterized by a slight swelling of the iris (mean score = 0.1429), and there were no changes in the cornea, conjunctiva, and chemosis (mean score = 0). However, the eye irritation can be fully recovered within the observation period of the irritation test results, which is 21 days. The test results are supported by the theory that pymetrozine has low acute toxicity and is classified as Toxicity category III in the eye irritation test for rabbits (slightly irritating) [25].

In addition, the insecticide formula with the active compound indoxacarb–emamectin benzoate (Endorse Plus 160/20 OD) at a dose of 0.1 mL which is administered topically to the eyes causes mild irritation to rabbit eyes compared to normal eyes (control), which is characterized by turbidity on the cornea (mean score = 0.0952), iris swelling (mean score = 0.4762), conjunctival redness (mean score = 0.0952), and chemosis (mean score = 0.0952 and 1482). However, the eye irritation can recover completely within the observation time of the irritation test results, which is 21 days. The test results are in accordance with the theory that indoxacarb has moderately irritating properties to the eyes of rabbits (classified as Toxicity category III). In addition, this can occur allegedly due to the mechanism of action of indoxacarb and emamectin benzoate, which are more toxic via the oral route compared to the topical route [30].

### 3.3. Skin Irritation Testing

Skin sensitization refers to an immune system response that causes an enhanced reaction to a chemical allergen. It happens when a vulnerable individual comes into contact with a chemical allergen, which leads to the development of sensitization. This allergen stimulates an immune response in the skin, resulting in the formation of contact sensitization. Sensitivity studies involve exposing test animals (such as guinea pigs) to the substance being tested to determine if there is a hypersensitivity effect [28]. This is assessed by comparing the scores obtained from exposing the animals to different concentrations of the substance during the induction period in the initial test, with the confirmation test period using control animals.

#### 3.3.1. Initial Test (Concentration Determination)

The initial test was carried out for each sample with varying concentrations of 12.5%, 25%, 50%, 75%, and 100% to determine concentrations that can cause erythema (for the induction phase) and concentrations that do not cause erythema (for the challenge phase).

Based on the test results, it was found that the highest concentrations of each insecticidal formula with the single active ingredient cypermethrin and the insecticidal formula with the compound active ingredient indoxacarb–emamectin benzoate which caused erythema were 100% and 50%. On the other hand, the insecticide formula with the single active ingredient pymetrozine did not induce erythema even at a concentration of 100%.

#### 3.3.2. Main Test

After the initial test, the induction test is carried out. The samples exposed to this phase were of 100% concentration (insecticide formula with the active ingredient cypermethrin), 100% concentration (insecticide formula with active pymetrozine), and 50% concentration (insecticide formula with active compound indoxacarb–emamectin benzoate).

Furthermore, the main test in skin sensitization testing is conducted on Day 27 using 3 guinea pigs to ensure that the observed reaction is indeed a sensitization response and not merely an irritation reaction. The samples that were exposed to the challenge phase were a concentration of 75% (cypermethrin), a concentration of 100% (pymetrozine), and a concentration of 25% (indoxacarb–emamectin benzoate).

**Figure 11:**
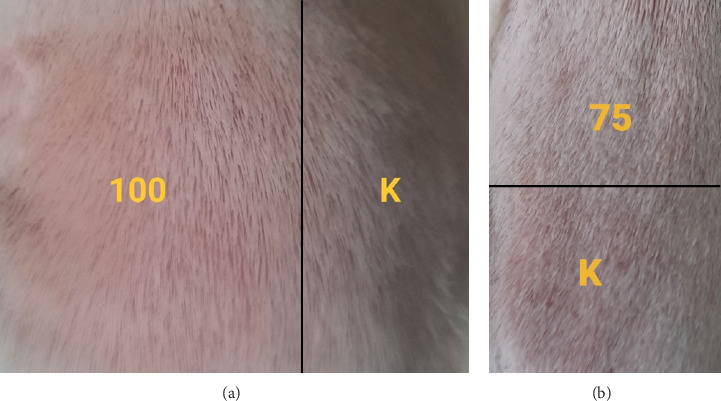
(a) Sensitization testing results of an insecticide formula containing 100% active ingredient cypermethrin on guinea pig skin (erythema observed in a small area). (b) Sensitization testing results of an insecticide formula containing 75% active ingredient cypermethrin on guinea pig skin (no observed changes).

According to Magnusson and Kligman [29], if the results of the skin sensitization test have a score ≥ 1, then it is categorized as a sensitizer. Based on the test results presented in Table 10, the insecticidal formula with the single active ingredient cypermethrin (Gusano 300 EC) applied topically to guinea pig skin caused a sensitization reaction characterized by erythema in a small area exposed to the sample (score = 1) compared to test area exposed to NaCl as a control (Figure 11). However, the erythema resolves completely within 48 h after the bandage is removed.

The insecticide formula with the single active ingredient pymetrozine (Vorum 50 WG), which was applied topically to guinea pig skin, did not or hardly caused a sensitization reaction, and no sign of erythema was seen in the test area exposed to the sample (score = 0) compared to the test area exposed to NaCl as a control.

Then, the insecticide formula with the active ingredient indoxacarb–emamectin benzoate (Endorse Plus 160/20 OD) applied topically to guinea pig skin caused a sensitization reaction characterized by erythema in a small portion of the test area exposed to the samples (score = 1) compared to the test area exposed to NaCl as a control. However, the erythema has not fully recovered within 48 h after the bandage is removed and completely recovered within 72 h after the bandage is removed. Further test results showed that exposure to indoxacarb–emamectin benzoate shows the minimum concentration of sensitization among the other two formulas. This can show that the formula is the most potent sensitizer compared to the other two formulas. Based on the tests, it can be concluded that cypermethrin is the second sensitizer after the insecticidal formula with the active compound indoxacarb–emamectin benzoate, while the single active insecticide formula pymetrozine is not a sensitizer (score = 0).

## 4. Discussion

This study successfully evaluated the skin irritation, eye irritation, and skin sensitization potential of three insecticide formulations: cypermethrin (Gusano 300 EC), pymetrozine (Vorum 50 WG), and indoxacarb–emamectin benzoate (Endorse Plus 160/20 OD). The findings indicate distinct toxicity profiles for each formulation, reflecting differences in their underlying pharmacological mechanisms.

The insecticide containing pymetrozine did not cause skin irritation or sensitization in guinea pigs and induced only mild eye irritation in rabbits. In contrast, cypermethrin caused minimal skin irritation, mild eye irritation, and sensitization reactions in guinea pigs, although these resolved within 48 h. The indoxacarb–emamectin benzoate formulation produced comparable mild eye irritation and more persistent skin sensitization, with reactions resolving within 72 h. These outcomes suggest that indoxacarb–emamectin benzoate is the most potent sensitizer among the tested formulations, followed by cypermethrin, while pymetrozine exhibits the lowest irritancy and sensitization potential.

Mechanistically, these differences may be explained by the distinct neurotoxic modes of action of the active ingredients. Cypermethrin, a Type II pyrethroid, prolongs the opening of voltage-gated sodium channels, potentially contributing to peripheral nerve hyperexcitability and localized inflammation [30]. Indoxacarb blocks sodium channels, and when combined with emamectin benzoate, a chloride channel modulator acting on GABA and glutamate-gated receptors, may produce synergistic ionic disruptions in mammalian tissues, potentially explaining the prolonged sensitization observed [31]. Meanwhile, pymetrozine operates by disrupting feeding behavior via chordotonal organ interference in insects, with no known direct action on mammalian ion channels, likely contributing to its low toxicity profile in this study [32].

Despite these findings, several important limitations must be noted. First, while the Magnusson and Kligman guinea pig maximization test (OECD 406) is a validated method for detecting sensitization, it does not provide EC3 values, an important metric for quantifying sensitization potency [33]. Future studies employing the local lymph node assay (LLNA) are recommended to estimate EC3 values and allow comparative potency classification. Second, histopathological analysis of the exposed tissues was not performed. Including microscopic evaluation in future work could better characterize the extent of cellular damage, inflammation, or tissue remodeling that may not be evident through gross observation alone. Third, although the outcomes were reported as mean values, inferential statistical analyses (e.g., ANOVA or Kruskal–Wallis tests) were not conducted due to the small sample size (*n* = 3 animals per group), which was determined based on ethical and logistical constraints in line with the replacement, reduction, and refinement (3R) principles. As a result, the data are interpreted descriptively, and conclusions regarding statistical significance should be approached cautiously. Future studies with larger sample sizes will allow for more robust statistical testing and validation of findings.

Moreover, the study design included only a negative control (NaCl 0.9%), and no validated positive control (e.g., sodium lauryl sulfate) was used. While OECD guidelines recommend the inclusion of positive controls to confirm system sensitivity, this was not feasible due to ethical and logistical limitations. Nonetheless, the observed irritation and sensitization responses suggest that the formulations possess measurable biological activity. Incorporating positive controls in future studies will be essential for benchmarking effect severity and confirming test system responsiveness. Lastly, a comprehensive toxicological risk assessment, including exposure modeling, repeated-dose studies, and margin of safety evaluations, was beyond the scope of this preliminary study. However, based on observed irritancy and sensitization, formulations containing cypermethrin and indoxacarb–emamectin benzoate may present a higher risk under occupational exposure. These findings highlight the importance of implementing adequate safety labeling, protective equipment use, and further regulatory evaluation. To strengthen the toxicological profile and safety recommendations, future research should include acute, subchronic, and chronic toxicity evaluations of all three formulations under various exposure scenarios.

## 5. Conclusion

This study presents one of the first comparative in vivo safety evaluations of commercially available insecticide formulations, cypermethrin (Gusano 300 EC), pymetrozine (Vorum 50 WG), and indoxacarb–emamectin benzoate (Endorse Plus 160/20 OD), conducted under standardized OECD guidelines (OECD 404, 405, and 406). The cypermethrin and indoxacarb–emamectin benzoate formulations exhibited minimal skin irritation, induced mild eye irritation, and triggered skin sensitization in guinea pigs. In contrast, the pymetrozine formulation did not cause skin irritation or sensitization and showed only mild eye irritation. Irritation reactions resolved within 14–21 days, while sensitization symptoms subsided within 48–72 h. These results provide important preliminary toxicological data for regulatory purposes and pesticide safety assessments. Future studies are encouraged to explore long-term dermal and ocular toxicity, optimize formulation components to reduce irritancy, and incorporate predictive modeling for occupational exposure risk. The novel comparative data generated by this study can serve as a valuable reference for improving pesticide safety and informing regulatory frameworks.

## Figures and Tables

**Table 1:** Draize dermal irritation scoring system [18, 19].

Skin reaction	Score
*Erythema and eschar formation*	
No erythema	0
Very slight erythema	1
Well-defined erythema	2
Moderate to severe erythema	3
Severe erythema (beet to crimson red) to slight eschar formation	4

*Edema formation*	
No edema	0
Very slight edema	1
Slight edema	2
Moderate edema	3
Severe edema	4

**Table 2:** Draize eye irritation scoring system [21].

Eye reaction	Value
*Cornea opacity*	
No opacity	0
Scattered or diffuse areas, details of the iris clearly visible	1
Easily discernible translucent area, details of the iris slightly obscured	2
Opalescent areas, no details of iris visible, size of pupil barely discernible	3
Opaque, iris invisible	4

*Iris*	
Normal	1
Folds above normal	2

*Conjunctive, redness*	
Vessel normal	0
Vessel definitely injected above normal	1
More diffuse, deeper crimson red, individual vessels not easily discernible	2
Diffuse beefy red	3

*Chemosis*	
No swelling	0
Any swelling above normal (including the nictitating membrane)	1
Swelling with lids about half closed	2
Swelling with lids about half closed to completely closed	3
Swelling with lids about more than half closed to completely closed	4

**Table 3:** Skin irritation scoring system [22].

Skin reaction	Value
No visible change	0
Discrete or patchy erythema	1
Moderate and confluent erythema	2
Intense erythema and swelling	3

**Table 4:** Skin irritation initial test result.

Sample	Irritation reaction	Score				
		Day 0	Day 2	Day 3	Day 7	Day 14
Cypermethrin	Erythema and eschar formation	1	1	1	1	1
	Edema formation	1	1	1	1	1

Pymetrozine	Erythema and eschar formation	1	1	1	1	1
	Edema formation	1	1	1	1	1

Indoxacarb–emamectin benzoate	Erythema and eschar formation	0	0	0	0	0
	Edema formation	0	0	0	0	0

**Table 5:** Skin irritation confirmation test result.

Sample	Rabbit	Irritation reaction	Score				
			Day 0	Day 2	Day 3	Day 7	Day 14
Cypermethrin	$\bar{x}$ 3 rabbit	Erythema and eschar formation	1	1	1	1	0
		Edema formation	0	0	0	0	0

Pymetrozine	$\bar{x}$ 3 rabbit	Erythema and eschar formation	0	0	0	0	0
		Edema formation	0	0	0	0	0

Indoxacarb–emamectin benzoate	$\bar{x}$ 3 rabbit	Erythema and eschar formation	1	1	1	1	0.333
		Edema formation	0	0	0	0	0

**Table 6:** Primary dermal irritation index calculation results.

Rabbit	Primary dermal irritation index		
	Cypermethrin	Pymetrozine	Indoxacarb–emamectin benzoate
1	0.83	0	1
2	0.83	0	0.83
3	0.83	0	1
4	0.83	0	0.83
Mean	0.83	0	0.915

**Table 7:** Eye irritation initial test result.

Sample	Irritation reaction	Score						
		Day 0	Day 1	Day 2	Day 3	Day 7	Day 14	Day 21
Cypermethrin	Cornea	1	1	2	2	1	0	0
	Iris	1	1	1	1	1	0	0
	Conjunctive	0	0	0	0	0	0	0
	Chemosis	2	3	3	0	0	0	0

Pymetrozine	Cornea	0	0	0	0	0	0	0
	Iris	0	0	0	0	0	0	0
	Conjunctive	0	0	0	0	0	0	0
	Chemosis	0	0	0	0	0	0	0

Indoxacarb–emamectin benzoate	Cornea	0	0	0	0	1	0	0
	Iris	1	1	1	1	1	0	0
	Conjunctive	0	0	0	0	0	0	0
	Chemosis	1	1	1	0	0	0	0

**Table 8:** Eye irritation confirmation test result.

Sample	Rabbit	Irritation reaction	Score						
			Day 0	Day 1	Day 2	Day 3	Day 7	Day 14	Day 21
Cypermethrin	$\bar{x}$ 2 rabbit	Cornea	2	1	1.5	1.5	1	0.5	0
		Iris	1	1	1	1	1	1	0.5
		Conjunctive	0	0	0	0	0.5	0	0
		Chemosis	2	2	1.5	1	1	1	0

Pymetrozine	$\bar{x}$ 2 rabbit	Cornea	0	0	0	0	0	0	0
		Iris	0	0	0.5	0	0.5	0.5	0
		Conjunctive	0	0	0	0	0	0	0
		Chemosis	0	0	0	0	0	0	0

Indoxacarb–emamectin benzoate	$\bar{x}$ 2 rabbit	Cornea	0.5	0	0	0	0	0	0
		Iris	1	0.5	0.5	0	0	0	0
		Conjunctive	0.5	0.5	0.5	0.5	0.5	0	0
		Chemosis	0	0	0	0	0	0	0

**Table 9:** Primary eye irritation index calculation result.

Irritation reaction	Primary eye irritation index		
	Cypermethrin	Pymetrozine	Indoxacarb–emamectin benzoate
Cornea	1.0476	0	0.0952
Iris	0.8095	0.1429	0.4762
Conjunctivae	0.0476	0	0.238
Chemosis	1.1905	0	0.1482

**Table 10:** Scoring of skin sensitization test results.

Day	Cypermethrin	Pymetrozine	Indoxacarb–emamectin benzoate
*Initial test (determining concentration)*			
0	Mild irritant concentration: 100% Nonirritant concentration: 75%	Mild irritant concentration: Nonirritant concentration: 100%	Mild irritant concentration: 50% Nonirritant concentration: 25%

*Induction test 1*			
0	1	0	1
1	1	0	1
2	0	0	1
6	1	0	1
7	1	0	1
8	0	0	1
13	1	0	1
14	1	0	1
15	0	0	0
27	0	0	0
28	0	0	0
29	0	0	0

*Induction test 2*			
0	1	0	1
1	1	0	1
2	0	0	1
6	1	0	1
7	1	0	1
8	0	0	1
13	1	0	1
14	1	0	1
15	0	0	0
27	0	0	0
28	0	0	0
29	0	0	0

*Induction test 3*			
0	1	0	1
1	1	0	1
2	0	0	1
6	1	0	1
7	1	0	1
8	0	0	1
13	1	0	1
14	1	0	1
15	0	0	0
27	0	0	0
28	0	0	0
29	0	0	0

*Note:* Description: 0 = no change; 1 = erythema in a small area (barely perceptible). Exposure for 6 h was conducted on Days 0, 6, 13, and 27.

## Data Availability

The data that support the findings of this study are available from the corresponding author upon reasonable request.
